# Resistance Training and Weight Loss in Older Adults: A Scoping Review

**DOI:** 10.1186/s40798-023-00613-4

**Published:** 2023-08-01

**Authors:** Andrew N. L. Buskard, Robert J. Petrella

**Affiliations:** 1 Department of Family Practice, Faculty of Medicine, University of British Columbia, Vancouver, BC Canada; 2 Centre for Studies in Family Medicine, Department of Family Medicine, Western University, London, ON Canada; 3 School of Kinesiology, Western University, London, ON Canada; 4 School of Kinesiology, Faculty of Education, University of British Columbia, Vancouver, BC Canada; 5 Department of Cardiovascular Medicine, Mayo Clinic and Foundation, Rochester, MN USA

**Keywords:** Older adults, Resistance training, Weight loss

## Abstract

Resistance training (RT) is one of the most effective interventions available to older adults wishing to slow the progressive loss of muscle size and strength known to occur with age. Less is known about the ability of RT to resist the onset of an equally problematic condition related to increased age: obesity. The objective of this scoping review was to characterize current research associated with RT and weight loss in older adults, including protocols, feasibility, and gaps in current knowledge. We searched six databases using variations of the terms "resistance training," "weight loss," and "older adults" for experimental or quasi-experimental studies published in the year 2009 or later. Studies were included if they featured at least one treatment group with a mean age of > 65 years that engaged in an RT-only exercise protocol with no aerobic or high-intensity interval component. Of the 6102 references identified by the initial database search, 24 were retained for analysis. Older women and older adults with obesity or sarcopenic obesity were the most studied groups (*n* = 13), followed by healthy community-dwelling older adults (*n* = 11) and studies involving older adults and some aspect of either dietary control or pharmaceutical intervention (*n* = 8). Significant between-study heterogeneity was observed in the RT characteristics researchers thought optimal for improving body composition measures in older adults. Changes in body composition, rather than total body mass, were found to be the essential variables to consider when evaluating the effectiveness of an RT intervention aimed at reducing chronic disease in older adults. Weight loss alone appears to be an incomplete and problematic outcome measure for older adults, with changes in body composition (ratio of fat mass to lean mass) being the more appropriate variable to emphasize in this population. However, it is important to note that only one study, showing questionably reproducible findings, found a significant lean body mass gain. The lack of abundant high-quality evidence demonstrating combined RT and a healthy diet can lead to significant fat loss and lean body mass gain, coupled with high attrition rates observed in many of the studies reviewed, highlight the need for further rigorous research.

## Key Points

- Changes in body composition, rather than total BM, are the essential variables to consider when evaluating the effectiveness of an RT intervention aimed at attenuating the onset of chronic disease in older adults
- Significant heterogeneity exists across the available literature regarding RT characteristics thought to be most effective at improving body composition measures in older adults.

## Introduction

While completely arresting the progression of strength and muscle mass losses with age is currently impossible, exercise provides a potent vehicle for significantly delaying the point at which they become a critical threat to an individual's capacity for independent living. Age-related loss of skeletal muscle mass and function, termed sarcopenia, is associated with physical frailty and increased risk of disability and morbidity. [1] Muscle mass losses typically begin in middle age at approximately − 1% per year, [1] and in severe instances, can accelerate to a total reduction of − 50% by the eighth decade of life. [2] Additionally, the rapidly aging population in most Western countries has increased risk factors for chronic disease, [3] in particular obesity, the rates of which have doubled in older adults since 1980 and continue to increase worldwide. [4] Obesity represents a clear and present danger to an individual's ability to grow healthfully into their later years. It can aptly be described as a 'gateway condition' that, if left unchecked, can lead to a constellation of debilitating chronic diseases such as hypertension, dyslipidemia, hyperlipidemia, type 2 diabetes, hyperglycemia and cardiovascular disease. [5] A logical extension of the high rates of obesity and sarcopenia currently present in older adults in developed countries is that a certain proportion of individuals will become affected by both conditions. Termed sarcopenic obesity (SO), the two contributing conditions (sarcopenia and obesity) often combine synergistically to create a more significant overall negative health impact than the total of the two combined [5].

Accordingly, the two highest priority outcomes for an obese older adult's exercise program are to increase LBM (thus attenuating sarcopenia) and decrease FM (thus resisting obesity). To date, substantial research has been conducted on how best to achieve the first objective in older adults, but little is known about how best to address the second, specifically how best to incorporate RT. The implication of this information for exercise professionals, and subsequently the reason for this review, is that exercise interventions for obese older adults that target indiscriminate reductions in BM may cause unintended harm if said losses result from a reduction in both FM *and* LBM. In other words, novel exercise interventions that facilitate significant reductions in BM in obese older adults are only valuable insofar as they are reductions in FM. This explains why body composition evaluations are a staple outcome measure in almost all applied exercise studies on this topic.

Despite an apparent recognition of this fact in the available literature, surprisingly few studies have investigated the optimal way to include RT in an exercise program focused on weight loss (WL) in obese older adults. The body of the literature addressing how best to incorporate RT into weight loss programs for older adults is sparse and heterogeneous concerning optimal parameters with which to apply it. Delineating the current state of knowledge on this topic is crucial as it will illuminate the areas most in need of study and provide an overall blueprint of enquiry for researchers to advance knowledge on the subject. Determining which modes of RT are likely to have the most significant effect and are also tolerable and feasible in older adults is an essential step toward improving the health of these individuals.

This scoping review aims to identify and characterize existing research on the use of RT exercise interventions as a modality to promote weight loss in older adults with the goal of knowledge translation and making recommendations about further areas of study. Specifically, we will describe populations that have been studied, how RT has been applied, what can be deduced about the feasibility and tolerability of RT used in said manner, what primary outcomes have been addressed and what gaps in the current knowledge have become evident.

## Methodological Framework

Scoping reviews can be creatively described in simple terms as the less-complex younger sibling of meta-analyses and systematic literature reviews. As the name suggests, scoping reviews are a way to obtain an initial 'lay of the land' of the existing literature on a given topic and often serve as precursors to more thorough and precisely defined meta-analysis or systematic review. [6, 7] Scoping reviews follow an established five-step framework, described elsewhere in summary [8] and expanded [7] form.

The research question was as follows: What is known in the literature about RT and WL in older adults, including dominant protocols, outcomes, feasibility and safety concerns, and what are the current gaps in the knowledge?

We searched six databases (Scopus, Medline, Embase, CINAHL, SPORTDiscus, and Google Scholar) for articles published up to May 2022. Search terms included combinations and variations of the following terms: "resistance training," "strength training," "weight loss," "older adult," and "senior." A description of the complete search strategy is included in supplemental materials (https://shorturl.at/mnsA4). These searches identified 6102 potential studies. Of these, 2671 references were removed as duplicates. Titles and, if necessary, the abstract of the 3431 remaining studies were revised for obviously disqualifying information such as the phrase "systematic review" in the title, a publication date prior to 2009, publication forum other than a peer-reviewed academic journal. Lastly, author AB conducted a full-text review of the remaining 909 studies against a checklist of established inclusion/exclusion criteria. Articles were required to score a perfect 6/6 and 4/4, respectively.

Inclusion criteria*:* (1) mean participant age > 65 years, or one mean cohort age > 65 years and not statistically different from the other groups; (2) experimental or semi-experimental design; (3) original source, peer-reviewed; (4) published as full-text in English; (5) exercise protocol was exclusively resistance training (no combined aerobic or HIT); and (6) weight loss a primary outcome measure. Exclusion criteria: (1) published before 2009; (2) review paper or not peer-reviewed; (3) did not use human subjects; and (4) mean age < 50 years.

Guided by the methodology reported in a recent scoping review on a similar topic by colleagues at a Canadian partner university, [9] eligible studies were grouped into four sub-groups for analysis. Data from these studies were extracted and charted by AB, including the population(s) studied, the study design, and the primary outcomes measured. Details of the RT protocol intervention were also charted. If noted, information related to participants' perceptions of exercise feasibility and tolerance was also extracted. Potential outcome variables recorded if present included attendance, adherence, dropouts/withdrawals, "enjoyability" or acceptance of protocol, and adverse events.

## Results

**Fig. 1:**
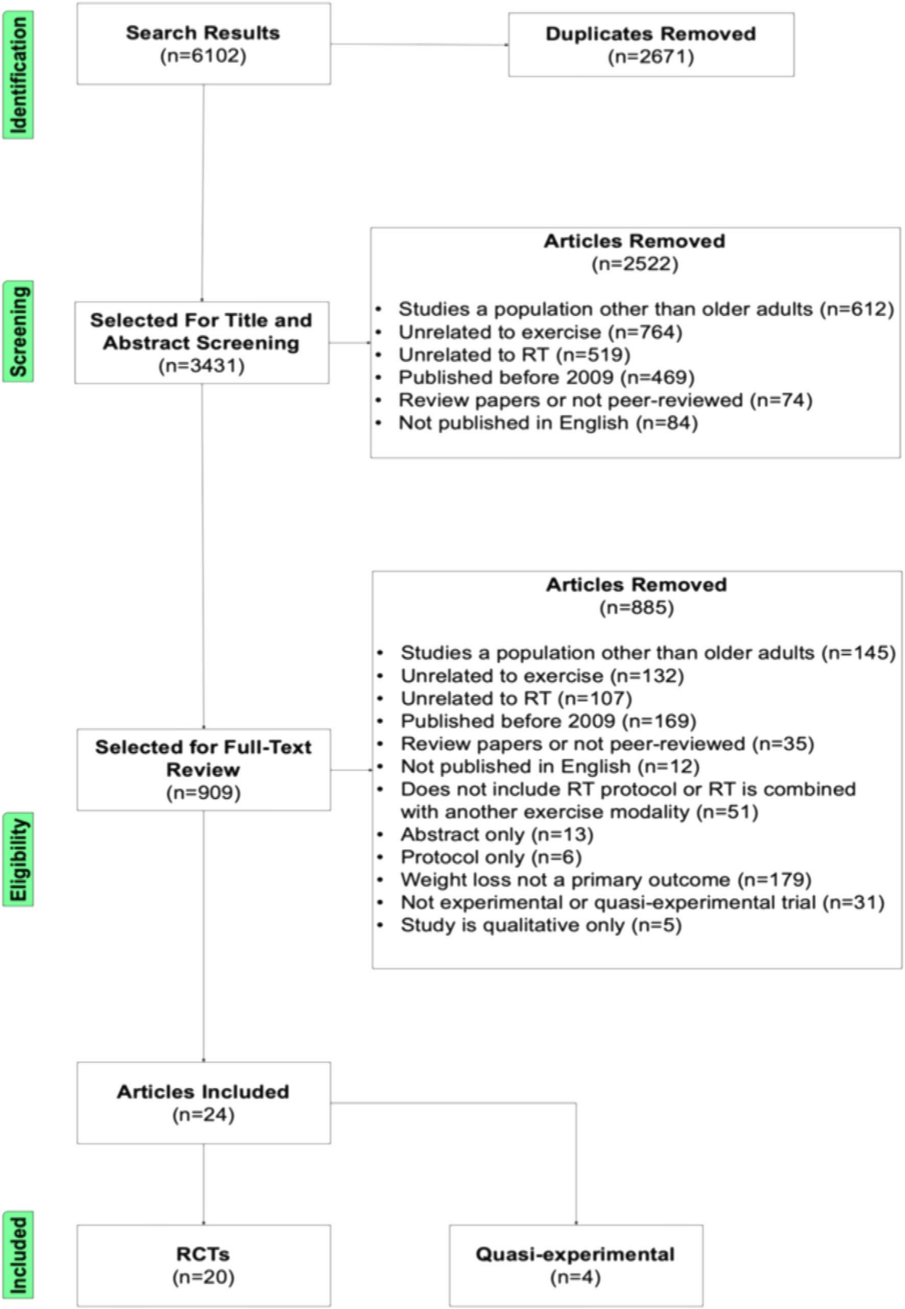
Flow chart of study selection procedures

The initial search yielded 6102 references. After removing 2671 duplicate studies and two screening stages, 24 studies were identified for inclusion in the analysis. Lack of orientation to exercise (*n* = 764) was one of the most common reasons for exclusion in the first screening phase, and a publication date before 2009 (*n* = 169) was one of the most common reasons for exclusion in the second screening phase. We present a detailed overview of the study selection procedure in Fig. 1.

Studies selected for inclusion were sub-divided according to the following key themes observed in the literature: studies involving healthy, community-dwelling older adults (*n* = 11), studies involving healthy, community-dwelling older women (*n* = 13), studies involving overweight, obese, or sarcopenic older adults (*n* = 13), and studies involving either dietary control or supplementation with a novel therapeutic drug (*n* = 8). We placed no limitations on the number of sub-groups to which a study could be allocated, provided the criteria for inclusion were satisfied. We report details about the experimental design, sample size, population(s), and baseline characteristics of the included studies in Table 1. Sample sizes ranged from 16 [10] to 451 [11] participants (mean [SD] = 96.5 [98.0]) and data from a total of 2438 individuals were considered across all studies. The mean ages of the study participants ranged from 60.0 [12] to 86.5 years [13] (mean [SD] = 67.1 [14.9] years). Twenty studies were randomized controlled, or crossover designs and 4 were quasi-experimental.

**Table 1:** Study design, sample size, and participant characteristics of included studies

Study	Study design	Population	Sample N	Age (years + SD)	Females N (%)
Aguiar et al. [18]	RCT; creatine supplementation vs. control	Healthy older women	18	64.9 (5.0)	18 (100)
Avila et al. [28]	RCT; DASH diet sedentary vs. Dash diet + MRT	Overweight and obese men and women	31	67.0 (4.0)	16 (51)
Balachandran et al. [32]	RCT; strength/hypertrophy vs. high-speed circuit training	Community-dwelling older adults with SO	21	71.8 (8.0)	16 (76)
Bardstu et al. [13]	RCT; RT vs. non-exercising control	Community and assisted-living older adults	104	86.5	64 (61)
Beavers et al. [29]	RCT; WL only vs. aerobic exercise training + WL vs. RT + WL	Obese, community-dwelling older adults	252	66.9 (4.7)	179 (71)
Bocalini et al. [30]	RCT; six arm based on BMI	Healthy, community-dwelling older women	78	65.3 (8.0)	78 (100)
Brochu et al. [15]	RCT; CR vs. RT + CR	Overweight and obese older women	137	58.0 (5.0)	137 (100)
Campbell et al. [10]	QE; Single cohort repeated measures	Postmenopausal, overweight women	16	68.0 (1.0)	16 (100)
Cavalcante et al. [26]	RCT; RT (3 × weekly) vs. RT (2 × weekly) vs. non-exercising control	Independent-living obese and overweight women	57	66.9 (5.3)	53 (100)
Chen et al. [33]	RCT; RT vs. CT vs. AT. Vs. non-exercising control	Community-dwelling older adults with SO	93	69.0 (3.1)	50 (54)
Coelho et al. [36]	RCT; NPRT vs. PRT. Vs. non-exercising control	Community-dwelling older women	42	67.0 (5.0)	42 (100)
Cunha et al. [27]	RCT; 1 set RT vs. 3 set RT vs. non-exercising control	Community-dwelling older women with SO	62	68.0 (4.3)	62 (100)
de Oliviera Silva et al. [3]	QE; 2 group design: participants with SO with vs. without SO	Community-dwelling older women with and without SO	49	> 60	49 (100)
Dib et al. [12]	RCT: 3 groups (various exercise order)	Community-dwelling older women	45	> 60	45 (100)
dos Santos et al. [14]	RCT: WPS RT vs. NPS RT vs. non-exercising control	Community-dwelling older women	59	67.3 (4.4)	59 (100)
Gadelha et al. [17]	RCT: RT vs. non-exercising control	Community-dwelling older women with SO	243	67.0 (5.2)	243 (100)
Gambassi et al. [8]	RCT: RT vs. non-exercising control	Community-dwelling older women	26	65.0 (3.0)	26 (100)
Gylling et al. [11]	RCT: heavy RT vs. moderate RT vs. non-exercising control	Community-dwelling older adults	451	66.0 (2.5)	275 (61)
Hanson et al. [37]	RCT; RT vs. non-exercising control	Healthy, sedentary older adults	81	70.5 (5.0)	16 (20)
Leenders et al. [24]	QE; 2 group RT program: men vs. women	Healthy, community-dwelling older adults	60	70.0 (1.0)	29 (48)
Shea et al. [34]	RCT 2 × 3 factorial; RT vs. no RT, pioglitazone	Older, non-diabetic men and women	88	69.8 (2.0)	40 (45)
Straight et al. [16]	QE; Single cohort repeated measures	Overweight and obese community-dwelling older adults	109	69.1 (6.2)	80 (73)
Verreijin et al. [31]	RCT; 2 × 2 factorial	Overweight and obese community-dwelling older adults	100	62.4 (5.4)	64 (64)
Vieira et al. [38]	RCT: two group RT differing by supervision style	Community-dwelling postmenopausal women	20	64.0 (3.7)	20 (100)

*1RM* one-repetition maximum, *ADP* air-displacement plethysmography, *AT* aerobic training, *BF* body fat, *BIA* bioelectrical impedance analysis, *BM* body mass, *BMI* body mass index, *CH* care home, *CR* caloric restriction, *CRP* creatine phosphate supplementation, *CT* combined training, *D* drug trial, *DASH* Dietary Approaches to Stop Hypertension, *DEXA* dual X-ray absorptiometry, *ES* effect size, *FFM* fat-free mass, *FM* fat mass, *H* healthy, *HRT* heavy resistance training, *MRT* moderate intensity resistance training, *NPRT* non-periodized resistance training, *NR* not reported, *NPRT* non-periodized resistance training, *O* overweight or obese, *P* postmenopausal, *PRT* periodized resistance training, *QE* quasi-experimental, *RCT* randomized-controlled trial, *RT* resistance training, *SO* sarcopenic obesity, *W* women only, *WL* weight loss, *WPS* wide pyramid system

Tables 2, 3, 4, 5 detail each study's exercise protocols and relevant WL-related outcome measures. Feasibility and tolerability information is provided if reported. Studies are grouped according to the four general categories of investigation present in the literature. Intervention lengths ranged from 8 weeks [14] to one year [11] at a frequency of either 2 or 3 training sessions per week in almost every case. Low homogeneity in RT modality utilized across all studies was observed, with methods ranging from elastic tubing and bodyweight exercises [13] to maximum-intensity efforts on computerized resistance exercise machines. [12] However, selectorized resistance exercise machines similar to those found in ordinary fitness centers were the most commonly utilized. Numerous methods were used to monitor exercise intensity, including but not limited to: specified percentages of the one-repetition maximum (1RM), [15] ratings of perceived exertion, [16] number of repetitions completed before failure [8] and prescribed entirely in advance by the study protocol. [17] Measures of feasibility and tolerability were rarely reported, in contrast with program adherence and dropout information, which was commonly reported and included values ranging from 52% dropout rate and 51% adherence [13] to 0 dropouts [18] and 94% adherence rate [14].

**Table 2:** RT studies in healthy, community-dwelling older adults

Study	Exercise protocol	WL-related outcome (s)	Feasibility/tolerability
Aguiar et al. [18]	Frequency: 3x/wk for 12 weeks Modality: Eight whole-body exercises primarily using selectorized exercise machines (2 sets of 10–15 repetitions per exercise per session)	No change in BM or %BF (via DEXA) in either group (CRP vs. placebo) relative to baseline. CRP group gained significantly more (*p* < 0.05) FFM (+ 3.2%) and muscle mass (+ 2.8%) than the placebo group over the course of the intervention. Results suggest creatine phosphate supplementation may be an effective dietary adjunct for older women participating in a structured RT program	Dropouts: 0 AEs: NR Tolerability: *see *Table 5 Feasibility: NR
Bardstu et al. [13]	Frequency: 2x/wk for 8 months Modality: Easily available, low-cost equipment such as elastic bands, body weight, and water canes aimed to strengthen the muscle groups most important for daily living activities; 2 sets of 10–12 repetitions (Weeks 1–5 progressing to 4 sets of 8–10 repetitions per exercise	No significant changes observed in BMI, %BF (via BIA), or FFM over the course of the study between the RT intervention group and the non-exercising control	Dropouts: 31 (52%) Adherence to intervention: 51% AEs: 0 Tolerability: medium-to-low based on high dropout rate and low adherence rate Feasibility: authors conclude their protocol is "feasible and possible to implement in real-life settings of older adults"
Coelho et al. [36]	Frequency: 2x/wk for 22 weeks Modality: Nine selectorized resistance exercise machines targeting whole-body muscle groups (3 sets of 8–12 repetitions per exercise, 'difficult' perceived intensity corresponding to a perceived exertion of 5–6 out of 10)	No significant differences in any body composition measure relative to baseline were observed in any group. Results indicate periodization strategies do not substantially influence the effectiveness of RT on WL on healthy, community-dwelling older adults	Dropouts: 6 (20%) Adherence to intervention: 89% AEs: 0 Tolerability: NR Feasibility: NR
de Oliviera Silva et al. [19]	Frequency: 2x/wk for 16 weeks Modality: Ten selectorized resistance exercise machines targeting whole-body muscle groups (3 sets per exercise progressing from 12–14 repetitions in Weeks 1–4 to 6–8 repetitions in Weeks 13–16)	Significant changes in post-intervention %BF (− 2.2%, *p* = 0.006; via DEXA) and FM (F = 5.22, *p* = 0.03) observed in non-SO group but not SO-group. Results suggest that adaptations induced by 16 weeks of RT are attenuated in elderly woman with SO, compromising improvements in adiposity indices and gains in LMM	Dropouts: 0 AEs: NR Tolerability: NR Feasibility: NR
Dib et al. [12]	Frequency: 3x/wk for 24 weeks Modality: Eight selectorized resistance exercise machines targeting whole-body muscle groups (3 sets of 10–15 repetitions in Weeks 1–12, 1 set each of 15, 10, 5 repetitions in Weeks 13–24)	Significant reduction in BF across all subjects in Weeks 1–12 (26.4 + 8.1 kg to 25.1 + 8.2 kg, *p* < 0.001; via DEXA). No significant change in BF across Weeks 13–24 for any group. Results suggest exercise order is not a major factor influencing the effectiveness of RT of WL in older women	Dropouts: 0 AEs: NR Tolerability: NR Feasibility: NR
dos Santos et al. [14]	Frequency: 3x/wk for 8 weeks Modality: Combination of free weights and machines targeting whole-body musculature (3 sets per exercise at either 12/10/8RM or 15/10/5RM	Significant (*p* < 0.05) improvements in body composition (total BF, android BF, gynoid BF) were observed in both intervention groups. Results suggest pyramidal loading strategies are not a primary determinant of RT program effectiveness in older women	Dropouts: 4 (7%) Adherence to intervention: 94% of participants completed > 85% of all exercise sessions AEs: 0 Tolerability: NR Feasibility: NR
Gambassi et al. [8]	Frequency: 2x/wk for 12 weeks Modality: Eight selectorized resistance exercise machines targeting whole-body muscle groups (3 sets at moderate intensity per exercise, aiming to reach temporary muscular failure at ~ 8 repetitions)	RT protocol led to significant (*p* < 0.05) improvements in FM (pre: 23.0 ± 1.2 kg vs. post: 20.0 ± 1.1 kg; via BIA) and FFM (pre: 38.0 ± 1.5 kg vs. post: 42.0 ± 1.4 kg; via BIA); relative to baseline. Results support existing literature that RT is an effective strategy during WL in older adults to attenuate concomitant loss of LBM	Dropouts: 0 AEs: NR Tolerability: NR Feasibility: NR
Gylling et al. [11]	Frequency: 3x/wk for 52 weeks Modality: Whole-body strength training program using either weight machines (HRT) or rubber bands and bodyweight (MRT). HRT group: three sets of 6–12 repetitions per exercise at approx. 70–85% 1RM. MRT group: three sets of 10–18 repetitions per exercise at approx. 50–60% 1RM	The HRT cohort demonstrated significant improvements in %BF and visceral FM relative to baseline (*p* < .0001; via DEXA), as well as to 12-month values in the MRT group (*p* < .01, ES: 0.41) and non-exercising control (*p* < .0001, ES: 0.53). Authors highlight the main finding that HRT appears to be more effective than MRT at eliciting improvements in LM and FM in older adults	Dropouts: 6 (1.9%) Adherence to intervention: 83% of participants completed training > 2x/wk AEs: NR Tolerability: NR Feasibility: NR
Hanson et al. [37]	*Phase 1* Frequency: 3x/wk for 10 weeks Modality: Unilateral training of the dominant leg knee extensor (near-maximal effort; pneumatic resistance machine) *Phase 2* Frequency: 3x/wk for 12 weeks Modality: Six pneumatic resistance exercise machines targeting whole-body muscle groups (one warm up set of 5 repetitions @ 50%1RM followed by 15 repetitions at 5RM load for each exercise). An additional 1–2 repetitions were completed immediately after the 5RM repetitions by reducing the load on the machine	Overall cohort (*p* < 0.01) and men (*p* < 0.05) demonstrated a significant increase in FFM (via DEXA) over the course of the study. Results support existing literature that RT is an effective strategy during WL in older adults to attenuate concomitant loss of LBM	Dropouts: 31 (38.8%) Adherence to intervention: 93.3 ± 1.3% (Phase 1), 87.6 ± 1.1% (Phase 2) AEs: pain and discomfort from pre-existing condition (n = 9) Tolerability: NR Feasibility: NR
Leenders et al. [24]	Frequency: 3x/wk for 6 months Modality: Selectorized resistance exercise machines. Leg-press and leg-extension: 4 sets of 10–15 repetitions at 60% 1RM (Week 1–4) progressing to 8 repetitions at 75–80% 1RM (Week 5 onward). Upper-body exercises: as above except always one less set	Significant post-intervention improvement in whole-body LM and FM (both via DEXA) was observed in both women (1.2 ± 0.2 kg, 5% ± 2%; *p* < .001) and men (1.2 ± 0.3 kg, 6% ± 1%;* p* < .001). Results suggest structured RT may equally benefit older men and women in terms of counteracting the loss of muscle mass and strength that occurs with age	Dropouts: 7 (11.6%) AEs: 2 cardiac events experienced away from study facility Tolerability: NR Feasibility: NR
Vieira et al. [38]	Frequency: 2x/wk for 16 weeks Modality: Eight selectorized resistance exercise machines targeting whole-body muscle groups. 2 sets per exercise; intensity: daily undulating between 12–14 RM, 10–12 RM, 8–10 RM, and 6–8 RM	Reduced BF and %BF (p < 0.05; via DEXA) were observed in the high-supervision group only. Authors conclude a greater supervision ratio during RT may induce more improvements in muscle strength and body composition than lower supervision ratio	Dropouts: 0 AEs: NR Tolerability: NR Feasibility: NR

*1RM* one-repetition maximum, *ADP* air-displacement plethysmography, *AT* aerobic training, *BF* body fat, *BIA* bioelectrical impedance analysis, *BM* body mass, *BMI* body mass index, *CH* care home, *CR* caloric restriction, *CRP* creatine phosphate supplementation, *CT* combined training, *D* drug trial, *DASH* Dietary Approaches to Stop Hypertension, *DEXA* dual X-ray absorptiometry, *ES* effect size, *FFM* fat-free mass, *FM* fat mass, *H* healthy, *HRT* heavy resistance training, *MRT* moderate intensity resistance training, *NPRT* non-periodized resistance training, *NR* not reported, *NPRT* non-periodized resistance training, *O* overweight or obese, *P* postmenopausal, *PRT* periodized resistance training, *QE* quasi-experimental, *RCT* randomized-controlled trial, *RT* resistance training, *SO* sarcopenic obesity, *W* women only, *WL* weight loss, *WPS* wide pyramid system

**Table 3:** RT studies in older women

Study	Exercise protocol	WL-related outcome(s)	Feasibility/tolerability
Aguiar et al. [18]	Frequency: 3x/wk for 12 weeks Modality: Eight whole-body exercises primarily using exercise machines (2 sets of 10–15 repetitions per exercise per session)	No change in BM or %BF (via DEXA) observed in either group (CRP vs. placebo) relative to baseline. CRP group gained significantly more (*p* < 0.05) FFM (+ 3.2%) and muscle mass (+ 2.8%) than the placebo group over the course of the study. Results suggest creatine phosphate supplementation may be an effective dietary adjunct for older women participating in a structured RT program	Dropouts: 0 AEs: NR Tolerability: *see *Table 5 Feasibility: NR
Bocalini et al. [30]	Frequency: 3x/wk for 12 weeks Modality: Whole body RT using elastic bands and free weights	Significant post-intervention reductions were observed in BM (overweight: − 4.5 ± 1.0%, obese: − 8.0 ± 0.8%), %BF (via skinfold analysis; overweight: − 11.0 ± 2.2%, obese: − 21.4 ± 2.1%) and FM (overweight: − 16.1 ± 3.2%, obese: − 31.2 ± 3.0%). No significant body composition changes were observed in participants with a healthy baseline BMI (18.5–24.9 kg/m^2). Results support existing literature regarding the beneficial effect of RT in obese and overweight women	Dropouts: 2 AEs: 0 Tolerability: NR Feasibility: NR
Brochu et al. [15]	Frequency: 3x/wk for 6 months Modality: Seven exercises targeting the whole body (leg press, chest press; lateral pull downs; shoulder press; arm curls, triceps extensions). 2–3 sets (15 repetitions, 65% 1RM) per exercise in Phase 1 progressing to 3–4 sets (10–12 repetitions, 75% 1RM) per exercise in Phase 4	Both CR and CR + RT were found to facilitate significant improvements in BM, %FM (via DEXA), and total FM. Results reinforce the complementary role exercise and diet play in healthy weight loss and upper limit of RT-alone to effect body composition	Dropouts: 30 (21.9%) AEs: 3 Tolerability: NR Feasibility: NR Adherence to intervention: 100%: *n* = 2, > 90% or more of the training sessions: *n* = 6
Campbell et al. [10]	Frequency: 3x/wk for 16 weeks Modality: Pneumatic resistance exercise machines targeting the whole body (2–3 sets of 8–12 repetitions per exercise @ approx. 80% 1RM)	RT did not lead to any additional in weight loss or %BF changes compared to the non-exercising control. Results reinforce the complementary role exercise and diet play in healthy weight loss and upper limit of RT-alone to effect body composition	Dropouts: 0 AEs: NR Tolerability: NR Feasibility: NR
Cavalcante et al. [26]	Frequency: 2x/wk vs. 3x/wk for 12 weeks Modality: Combination of machines and free weights targeting whole body muscle groups (one set of 10–15 repetitions per exercise)	Both exercise frequencies (2 × weekly, 3 × weekly) led to significant (*p* < 0.05) reductions in %BF (–1.7%, –2.7%, respectively; via DEXA) over the course of the study. No significant changes in BM were observed in either RT group or the non-exercising control. Results suggest 2 days per week may be the optimal RT frequency for obese older women	Dropouts: 4 (9.5%) AEs: NR Tolerability: NR Feasibility: NR
Coelho et al. [36]	Frequency: 2x/wk for 22 weeks Modality: Nine selectorized resistance exercise machines targeting whole-body muscle groups (3 sets of 8–12 repetitions per exercise, 'difficult' perceived intensity corresponding to a perceived exertion of 5–6 out of 10)	No significant differences in any body composition measure relative to baseline were observed in any group. Results indicate periodization strategies do not substantially influence the effectiveness of RT on WL on healthy, community-dwelling older adults	Dropouts: 6 (20%) Adherence to intervention: 89% AEs: 0 Tolerability: NR Feasibility: NR
Cunha et al. [27]	Frequency: 3x/wk for 12 weeks Modality: Eight selectorized resistance exercise machines targeting whole-body muscle groups (either 1 set or 3 sets of 10–15 repetitions per exercise, depending on intervention group)	Significant reductions in %BF (− 6.3%, *p* < 0.05; via DEXA) were observed in the 3 sets/exercise group but not the 1 set/exercise group or non-exercising control. Results indicate higher training volumes of RT may lead to greater improvements in body composition	Dropouts: 5 (10/8%) Adherence to intervention: ≥ 85% of the total sessions for all participants AEs: NR Tolerability: NR Feasibility: NR
de Oliviera Silva et al. [19]	Frequency: 2x/wk for 16 weeks Modality: Ten selectorized resistance exercise machines targeting whole-body muscle groups (3 sets per exercise progressing from 12–14 repetitions in Weeks 1–4 to 6–8 repetitions in Weeks 13–16)	Significant changes in post-intervention %BF (− 2.2%, *p* = 0.006; via DEXA) and FM (F = 5.22, *p* = 0.03) were observed in non-SO group but not SO-group. Results suggest that adaptations induced by 16 weeks of RT are attenuated in elderly woman with SO, compromising improvements in adiposity indices and gains in LMM	Dropouts: 0 AEs: NR Tolerability: NR Feasibility: NR
Dib et al. [12]	Frequency: 3x/wk for 24 weeks Modality: Eight selectorized resistance exercise machines targeting whole-body muscle groups (3 sets of 10–15 repetitions in Weeks 1–12, 1 set each of 15, 10, 5 repetitions in Weeks 13–24)	Significant reduction in BF across all subjects in Weeks 1–12 (26.4 + 8.1 kg to 25.1 + 8.2 kg, *p* < 0.001; via DEXA). No significant change in BF across Weeks 13–24 for any group. Results suggest exercise order is not a major factor influencing the effectiveness of RT of WL in older women	Dropouts: 0 AEs: NR Tolerability: NR Feasibility: NR
dos Santos et al. [14]	Frequency: 3x/wk for 8 weeks Modality: Combination of free weights and machines targeting whole-body musculature (3 sets per exercise at either 12/10/8RM or 15/10/5RM	Significant (*p* < 0.05) improvements in body composition (total BF, android BF, gynoid BF) were observed in both intervention groups. Results suggest pyramidal loading strategies are not a primary determinant of RT program effectiveness in older women	Dropouts: 4 (7%) Adherence to intervention: 94% of participants completed > 85% of all exercise sessions AEs: 0 Tolerability: NR Feasibility: NR
Gadelha et al. [17]	Frequency: 3x/wk for 24 weeks Modality: Eight selectorized resistance exercise machines targeting whole-body muscle groups (3 sets progressing from 12 repetitions at 60% 1RM in Weeks 1–4 to 8 repetitions at 80% 1RM in weeks 9–12)	Significant increase in fat-free mass (0.6 + 0.15 kg, *p* < 0.01), but no change in BM or %BF (all via DEXA). Authors conclude RT is an effective approach to promote body composition alterations in older women, particularly those with SO	Dropouts: 0 AEs: NR Tolerability: NR Feasibility: NR
Gambassi et al. [8]	Frequency: 2x/wk for 12 weeks Modality: Eight selectorized resistance exercise machines targeting whole-body muscle groups (3 sets at moderate intensity per exercise, aiming to reach temporary muscular failure at ~ 8 repetitions)	RT protocol led to significant (*p* < 0.05) improvements in FM (pre: 23.0 ± 1.2 kg vs. post: 20.0 ± 1.1 kg; via BIA) and FFM (pre: 38.0 ± 1.5 kg vs. post: 42.0 ± 1.4 kg; via BIA); relative to baseline. Results support existing literature that RT is an effective strategy during WL in older adults to attenuate concomitant loss of LBM	Dropouts: 0 AEs: NR Tolerability: NR Feasibility: NR
Vieira et al. [38]	Frequency: 2x/wk for 16 weeks Modality: Eight selectorized resistance exercise machines targeting whole-body muscle groups. 2 sets per exercise; intensity: daily undulating between 12–14 RM, 10–12 RM, 8–10 RM, and 6–8 RM	Reduced BF and %BF (p < 0.05; via DEXA) were observed in the high-supervision group only. Authors conclude a greater supervision ratio during RT may induce more improvements in muscle strength and body composition than lower supervision ratio	Dropouts: 0 AEs: NR Tolerability: NR Feasibility: NR

*1RM* one-repetition maximum, *ADP* air-displacement plethysmography, *AT* aerobic training, *BF* body fat, *BIA* bioelectrical impedance analysis, *BM* body mass, *BMI* body mass index, *CH* care home, *CR* caloric restriction, *CRP* creatine phosphate supplementation, *CT* combined training, *D* drug trial, *DASH* Dietary Approaches to Stop Hypertension, *DEXA* dual X-ray absorptiometry, *ES* effect size, *FFM* fat-free mass, *FM* fat mass, *H* healthy, *HRT* heavy resistance training, *MRT* moderate intensity resistance training, *NPRT* non-periodized resistance training, *NR* not reported, *NPRT* non-periodized resistance training, *O* overweight or obese, *P* postmenopausal, *PRT* periodized resistance training, *QE* quasi-experimental, *RCT* randomized-controlled trial, *RT* resistance training, *SO* sarcopenic obesity, *W* women only, *WL* weight loss, *WPS* wide pyramid system

**Table 4:** ﻿RT studies in obesity/sarcopenic obesity

Study	Exercise protocol	WL-related outcome (s)	Feasibility/tolerability
Avila et al. [28]	Frequency: 3x/wk for 10 weeks Modality: selectorized resistance exercise machines (six upper and lower body; 4 sets × 8–12 repetitions) + stretching	Significant reductions relative to baseline were observed in BM (− 3.3 + 0.8 kg, *p* < 0.001) and FM (− 4.1 + 0.9 kg, *p* < 0.001) in the DASH + RT group, while significant reductions in BM were observed in the DASH-only group (− 1.7 + 0.9 kg, *p* = 0.044). Results support existing knowledge that exercise + diet approaches to WL are generally more effective than diet-only approaches	Dropouts: 1 (96% adherence) AEs: 1 × minor hip extensor strain Tolerability: NR Feasibility: NR
Balachandran et al. [32]	Frequency: 2x/wk for 15 weeks Modality: 5 lower body and 6 upper body pneumatic exercise machines (3 sets of 10–12 repetitions using 70% of their 1RM)	No significant between or within-group differences were observed on any body composition outcome measure	Dropouts: 1 Adherence to intervention: 85% AEs: 1 × shoulder pain due to pre-existing injury Tolerability: NR Feasibility: NR
Beavers et al. [29]	Frequency: 4x/wk (2 × upper body, 2 × lower body) for 18 months Modality: eight selectorized resistance exercise machines (3 sets of 10–12 repetitions)	Total BM loss was greatest when WL was combined with exercise (WL: − 5.7 + 0.7 kg, WL + AT: − 8.5 + 0.7 kg, WL + RT: − 8.7 + 0.7 kg; *p* < 0.01). LM loss was greatest in WL + AT (− 1.6 + 0.3 kg, − 3.1%) compared with WL + RT (− 0.8 + 0.3 kg, − 1.5%) or WL (− 1.0 + 0.3 kg; − 2.0%); both *p* = 0.02. Results confirm existing literature WL + RT is preferable to WL + AT with respect to exercise aimed at improving body composition in obese and overweight older adults	Dropouts: 14 (17%) AEs: 2 × dropout due to medical complication Tolerability: NR Feasibility: NR
Bocalini et al. [30]	Frequency: 3x/wk for 12 weeks Modality: whole body RT using elastic bands and free weights	Significant post-intervention reductions were observed in BM (overweight: − 4.5 ± 1.0%, obese: − 8.0 ± 0.8%), %BF (via skinfold analysis; overweight: − 11.0 ± 2.2%, obese: − 21.4 ± 2.1%) and FM (overweight: − 16.1 ± 3.2%, obese: − 31.2 ± 3.0%). No significant body composition changes were observed in participants with a healthy baseline BMI (18.5–24.9 kg/m^2). Results support existing literature regarding the beneficial effect of RT in obese and overweight women	Dropouts: 2 AEs: 0 Tolerability: NR Feasibility: NR
Brochu et al. [15]	Frequency: 3x/wk for 6 months Modality: Seven exercises targeting the whole body (leg press, chest press; lateral pull downs; shoulder press; arm curls, triceps extensions). 2–3 sets (15 repetitions, 65% 1RM) per exercise in Phase 1 progressing to 3–4 sets (10–12 repetitions, 75% 1RM) per exercise in Phase 4	Both CR and CR + RT were found to facilitate significant improvements in BM, %FM (via DEXA), and total FM. Results reinforce the complementary role exercise and diet play in healthy weight loss and upper limit of RT-alone to effect body composition	Dropouts: 30 (21.9%) AEs: 3 Tolerability: NR Feasibility: NR Adherence to intervention: *see *Table 3
Campbell et al. [10]	Frequency: 3x/wk for 16 weeks Modality: Pneumatic resistance exercise machines targeting the whole body (2–3 sets of 8–12 repetitions per exercise @ approx. 80% 1RM)	RT did not lead to any additional in weight loss or %BF changes compared to the non-exercising control. Results reinforce the complementary role exercise and diet play in healthy weight loss and upper limit of RT-alone to effect body composition	Dropouts: 0 AEs: NR Tolerability: NR Feasibility: NR
Cavalcante et al. [26]	Frequency: 2x/wk vs. 3x/wk for 12 weeks Modality: Combination of machines and free weights targeting whole body muscle groups (one set of 10–15 repetitions per exercise)	Both exercise frequencies (2 × weekly, 3 × weekly) led to significant (*p* < 0.05) reductions in %BF (–1.7%, –2.7%, respectively; via DEXA) over the course of the study. No significant changes in BM were observed in either RT group or the non-exercising control. Results suggest 2 days per week may be the optimal RT frequency for obese older women	Dropouts: 4 (9.5%) AEs: NR Tolerability: NR Feasibility: NR
Chen et al. [33]	Frequency: 2x/wk (Week 1–8), 0x/wk (Week 9–12) Modality: Selectorized weight training machines targeting large systemic muscle groups (shoulder press, bicep curl, triceps curl, bench press, deadlift, leg swing, squat, standing row, unilateral row, and split front squat) at 60–70% 1RM (three sets of 8–12 repetitions)	Total body weight and FM significantly lower than non-exercising control at Weeks 8 and 12 (*p* < 0.05). No significant differences in any body composition measure at study conclusion between RT-only group and AT-only and AT-AT combined groups	Dropouts: 7 (31.8%) AEs: NR Tolerability: NR Feasibility: NR
Cunha et al. [27]	Frequency: 3x/wk for 12 weeks Modality: Eight selectorized resistance exercise machines targeting whole-body muscle groups (either 1 set or 3 sets of 10–15 repetitions per exercise, depending on intervention group)	Significant reductions in %BF (− 6.3%, *p* < 0.05; via DEXA) were observed in the 3 sets/exercise group but not the 1 set/exercise group or non-exercising control. Results indicate higher training volumes of RT may lead to greater improvements in body composition	Dropouts: 5 (10/8%) Adherence to intervention: ≥ 85% of the total sessions for all participants AEs: NR Tolerability: NR Feasibility: NR
de Oliviera Silva et al. [19]	Frequency: 2x/wk for 16 weeks Modality: Ten selectorized resistance exercise machines targeting whole-body muscle groups (3 sets per exercise progressing from 12–14 repetitions in Weeks 1–4 to 6–8 repetitions in Weeks 13–16)	Significant changes in post-intervention %BF (− 2.2%, *p* = 0.006; via DEXA) and FM (F = 5.22, *p* = 0.03) observed in non-SO group but not SO-group. Results suggest that adaptations induced by 16 weeks of RT are attenuated in elderly woman with SO, compromising improvements in adiposity indices and gains in LMM	Dropouts: 0 AEs: NR Tolerability: NR Feasibility: NR
Gadelha et al. [17]	Frequency: 3x/wk for 24 weeks Modality: Eight selectorized resistance exercise machines targeting whole-body muscle groups (3 sets progressing from 12 repetitions at 60% 1RM in Weeks 1–4 to 8 repetitions at 80% 1RM in weeks 9–12)	Significant increase in fat-free mass (0.6 + 0.15 kg, *p* < 0.01), but no change in BM or %BF (all via DEXA). Authors conclude RT is an effective approach to promote body composition alterations in older women, particularly those with SO	Dropouts: 0 AEs: NR Tolerability: NR Feasibility: NR
Straight et al. [28]	Frequency: 2x/wk for 8 weeks Modality: Combination of free weights, elastic tubing and ankle weights targeting whole body muscle groups (three sets of 8–12 repetitions per exercise)	Exercise intervention facilitated significant improvements in BM (–1.0 ± 1.8 kg, *p* < 0.001), BMI (–0.4 ± 0.8 kg/m^2^, *p* < 0.001; via BIA), %FM (–0.5 ± 1.4%, *p* < 0.001; via BIA), and FM (–0.8 ± 1.6 kg, *p* < 0.001; via BIA) relative to baseline	Dropouts: 14 (12.8%) AEs: 1 (strained muscle leading to dropout Tolerability: potentially enhanced by use of RPE to monitor RT intensity [39] Feasibility: enhanced by use of portable and inexpensive RT modalities such as ankle weights, elastic tubing
Verreijin et al. [31]	Frequency: 3x/wk for 10 weeks Modality: Squats, lunges, chest press, shoulder press, biceps curls, triceps extensions, standing rows, step-ups and crunches (2 sets per exercise progressing to 3 sets)	Significant decrease in BM and FM across all groups (*p* < 0.05; both via ADP), highest in exercise cohorts although the between-group difference was non-significant. There was no significant effect of high protein and exercise on change in FFM and FM, but RT significantly decreased body fat percentage with 0.8% (*p* = 0.048)	Dropouts: 32 (32%) Adherence to intervention: mean adherence = 2.8 ± 0.3 times/week AEs: 1 Tolerability: low considering high dropout rate Feasibility: low considering degree of exercise supervision and dietary control required

*1RM* one-repetition maximum, *ADP* air-displacement plethysmography, *AT* aerobic training, *BF* body fat, *BIA* bioelectrical impedance analysis, *BM* body mass, *BMI* body mass index, *CH* care home, *CR* caloric restriction, *CRP* creatine phosphate supplementation, *CT* combined training, *D* drug trial, *DASH* Dietary Approaches to Stop Hypertension, *DEXA* dual X-ray absorptiometry, *ES* effect size, *FFM* fat-free mass, *FM* fat mass, *H* healthy, *HRT* heavy resistance training, *MRT* moderate intensity resistance training, *NPRT* non-periodized resistance training, *NR* not reported, *NPRT* non-periodized resistance training, *O* overweight or obese, *P* postmenopausal, *PRT* periodized resistance training, *QE* quasi-experimental, *RCT* randomized-controlled trial, *RT* resistance training, *SO* sarcopenic obesity, *W* women only, *WL* weight loss, *WPS* wide pyramid system

**Table 5:** RT + supplement or dietary control studies

Study	Exercise protocol	WL-related outcome(s)	Feasibility/tolerability
Avila et al. [28]	Frequency: 3x/wk for 10 weeks Modality: selectorized resistance exercise machines (six upper and lower body; 4 sets × 8–12 repetitions) + stretching	Significant reductions relative to baseline were observed in BM (− 3.3 + 0.8 kg, *p* < 0.001) and FM (− 4.1 + 0.9 kg, *p* < 0.001) in the DASH + RT group, while significant reductions in BM were observed in the DASH-only group (− 1.7 + 0.9 kg, *p* = 0.044). Results support existing knowledge that exercise + diet approaches to WL are generally more effective than diet-only approaches	Dropouts: 1 (96% adherence) AEs: 1 × minor hip extensor strain Tolerability: NR Feasibility: NR
Aguiar et al. [18]	Frequency: 3x/wk for 12 weeks Medication: Creatine monohydrate (5.0 g/day vs. placebo) WL outcome measure: Δ body mass, Δ %BF using DEXA	No change in BM or %BF (via DEXA) observed in either group (CRP vs. placebo) relative to baseline. CRP group gained significantly more (*p* < 0.05) FFM (+ 3.2%) and muscle mass (+ 2.8%) than the placebo group over the course of the study. Results suggest creatine phosphate supplementation may be an effective dietary adjunct for older women participating in a structured RT program	Dropouts / AEs: *see *Table 2 Tolerability: CR supplementation appears to increase tolerable volume of RT Feasibility: *see *Table 2
Beavers et al. [29]	Frequency: 4x/wk (2 × upper body, 2 × lower body) for 18 months Modality: eight selectorized resistance exercise machines (3 sets of 10–12 repetitions)	Total BM loss was greatest when WL was combined with exercise (WL: − 5.7 + 0.7 kg, WL + AT: − 8.5 + 0.7 kg, WL + RT: − 8.7 + 0.7 kg; *p* < 0.01). LM loss was greatest in WL + AT (− 1.6 + 0.3 kg, − 3.1%) compared with WL + RT (− 0.8 + 0.3 kg, − 1.5%) or WL (− 1.0 + 0.3 kg; − 2.0%); both *p* = 0.02. Results confirm and existing literature WL + RT is preferable to WL + AT with respect to exercise aimed at improving body composition in obese and overweight older adults	Dropouts: 14 (17%) AEs: 2 × dropout due to medical complication Tolerability: NR Feasibility: NR
Brochu et al. [15]	Frequency: 3x/wk for 6 months Modality: Seven exercises targeting the whole body (leg press, chest press; lateral pull downs; shoulder press; arm curls, triceps extensions). 2–3 sets (15 repetitions, 65% 1RM) per exercise in Phase 1 progressing to 3–4 sets (10–12 repetitions, 75% 1RM) per exercise in Phase 4	Both CR and CR + RT were found to facilitate significant improvements in BM, %FM (via DEXA), and total FM. Results reinforce the complementary role exercise and diet play in healthy weight loss and upper limit of RT-alone to effect body composition	Dropouts: 30 (21.9%) AEs: 3 Tolerability: NR Feasibility: NR Adherence to intervention: *see *Table 3
Campbell et al. [10]	Frequency: 3x/wk for 16 weeks Modality: Pneumatic resistance exercise machines targeting the whole body (2–3 sets of 8–12 repetitions per exercise @ approx. 80% 1RM)	RT did not lead to any additional in weight loss or %BF changes compared to the non-exercising control. Results reinforce the complementary role exercise and diet play in healthy weight loss and upper limit of RT-alone to effect body composition	Dropouts: 0 AEs: NR Tolerability: NR Feasibility: NR
Shea et al. [34]	Frequency: 3x/wk for 16 weeks Modality: 2 × pneumatic resistance exercise machines (lower body) + combination of Nautilus resistance machines and dumbbells (upper body). Progression for all exercises was as follows. Week 1: two sets of 8–10 reps at 40–50% of 1RM; Week 2: three sets of 8–10 reps at 50–60% of 1RM; Weeks 3–16: three sets of 8–10 reps at 70% of 1RM	Men who were given pioglitazone lost more visceral abdominal fat than men who were not given pioglitazone (*p* = 0.007), while women who were given pioglitazone lost less thigh subcutaneous fat (*p* = 0.002). RT diminished thigh muscle loss in men and women (RT vs. no RT men: *p* = 0.005, women: *p* = 0.04). In overweight/obese older men undergoing weight loss, pioglitazone increased visceral fat loss and RT reduced skeletal muscle loss	Dropouts: 7 (8%) AEs: 0 Tolerability: Feasibility
Straight et al. [28]	Frequency: 2x/wk for 8 weeks Modality: Combination of free weights, elastic tubing and ankle weights targeting whole body muscle groups (three sets of 8–12 repetitions per exercise)	Significant reductions observed in BM (–1.0 ± 1.8 kg, *p* < 0.001), BMI (–0.4 ± 0.8 kg/m^2^, *p* < 0.001; via BIA), %FM (–0.5 ± 1.4%, *p* < 0.001; via BIA), and FM (–0.8 ± 1.6 kg, *p* < 0.001; via BIA)	Dropouts: 14 (12.8%) AEs: 1 (strained muscle leading to dropout Tolerability: potentially enhanced by use of RPE to monitor RT intensity [39] Feasibility: enhanced by use of portable and inexpensive RT modalities such as ankle weights, elastic tubing
Verreijin et al. [31]	Frequency: 3x/wk for 10 weeks Modality: Squats, lunges, chest press, shoulder press, biceps curls, triceps extensions, standing rows, step-ups and crunches (2 sets per exercise progressing to 3 sets)	Significant decrease in BM and FM across all groups (*p* < 0.05; both via ADP), highest in exercise cohorts although the between-group difference was non-significant. There was no significant effect of high protein and exercise on change in FFM and FM, but RT significantly decreased body fat percentage with 0.8% (*p* = 0.048)	Dropouts: 32 (32%) Adherence to intervention: mean adherence = 2.8 ± 0.3 times/week AEs: 1 Tolerability: low considering high dropout rate Feasibility: low considering degree of exercise supervision and dietary control required

*1RM* one-repetition maximum, *ADP* air-displacement plethysmography, *AT* aerobic training, *BF* body fat, *BIA* bioelectrical impedance analysis, *BM* body mass, *BMI* body mass index, *CH* care home, *CR* caloric restriction, *CRP* creatine phosphate supplementation, *CT* combined training, *D* drug trial, *DASH* Dietary Approaches to Stop Hypertension, *DEXA* dual X-ray absorptiometry, *ES* effect size, *FFM* fat-free mass, *FM* fat mass, *H* healthy, *HRT* heavy resistance training, *MRT* moderate intensity resistance training, *NPRT* non-periodized resistance training, *NR* not reported, *NPRT* non-periodized resistance training, *O* overweight or obese, *P* postmenopausal, *PRT* periodized resistance training, *QE* quasi-experimental, *RCT* randomized-controlled trial, *RT* resistance training, *SO* sarcopenic obesity, *W* women only, *WL* weight loss, *WPS* wide pyramid system

### Healthy, Community-Dwelling Older Adults

Studies included in this sub-group (*n* = 11) investigated various RT approaches to WL and, more importantly, fat mass (FM) reduction while preserving lean muscle mass (LMM) in community-dwelling older adults with no overt co-morbidity (Table 2**).** All studies except one [13] utilized selectorized resistance exercise machines, and common to all investigations was the recognition that WL alone is an inappropriate marker of RT effectiveness in this population and that RT interventions leading to reduced BM are only of value if researchers can demonstrate LBM was preserved in the process. Accordingly, the gold standard for a RT protocol designed for healthy older adults is one that is oriented to changes in *body composition*, where FM decreases, LMM increases, and total BM might remain unchanged.

An investigation that appears to have most effectively reached this target is that of Gambassi et al. [8], in which the researchers' experimental RT protocol was able to facilitate both a significant increase in FFM (pre: 38.0 ± 1.5 kg vs. post: 42.0 ± 1.4 k, *p* < 0.05) and a significant decrease in FM (pre: 23.0 ± 1.2 kg vs. post: 20.0 ± 1.1 kg, *p* < 0.05) in a cohort of older adults performing 12 weeks of twice-weekly RT. Of particular note was the researchers' decision to utilize repetitions performed to temporary muscular failure and a high overall exercise intensity (80% maximum load) at this population's upper recommended [19] limit. It must be noted, however, that the author’s observed LBM increase of approximately four kilograms in the experimental group over the 12-week intervention is significantly higher than those reported in similar studies [40, 41]. Given the relatively low sample size of the author’s intervention group (*n* = 13), it may be prudent to consider this response magnitude an outlier until an appropriately powered replication study can be conducted.

### Older Women

Similar to RT programming for healthy older adults discussed above, the guiding principle underlining the gold standard of RT programs designed for older women is not the attainment of BM losses as a sole end goal, but rather the achievement of reductions in FM while maintaining or increasing LBM [10, 12, 17, 20, 21]. The major distinction between the previous sub-group and the current population is the need to ensure, at minimum, the maintenance of LBM in structured exercise programs is elevated in older women compared to older men. [21, 22] The relative loss of muscle mass and strength with age has been reported to be similar for men and women. [23] However, the loss of LBM and strength may represent a more significant health concern for women. [24] Compared to men, women typically have more frequent and severe problems with sarcopenia, functional capacity, frailty and disability. [25]

We evaluated 13 RT studies examining WL and body composition changes in older women (Table 3). Heterogeneity concerning RT modality, intervention duration and frequency, body composition, feasibility, tolerance, and adherence mirrored those found in the total cohort. One particular outcome of interest is the finding from Brochu et al.’s [15] and Campbell et al.’s [10] investigations of RT performed in conjunction with dietary restriction in which an upper limit was detected for the ability of RT-alone to mediate meaningful changes in body composition. Their findings underscore the fundamental principle that diet and exercise must be considered in conjunction to create the most effective intervention for older women. A particular outcome of interest is that there may be no additional benefit on body composition outcomes from a 3x/weekly RT program compared to a 2x/weekly program. [26] Two other particular outcomes of note are creatine phosphate supplementation may be an effective pharmaceutical strategy to maximize LBM responses to RT, [18] and higher training volumes may lead to more significant improvements in body composition. [27]

### Sarcopenia and Obesity

Older adults face both an elevated risk of developing sarcopenia as they age as well as developing obesity; a proportion of individuals will develop both conditions. Rather than the overall health impact of sarcopenic obesity equaling the sum of the two contributing conditions combined, a synergistic negative effect can often occur, leading to an overall health impact greater than the individual sum of its parts. [5] Persons with concomitant sarcopenia and obesity face the combined metabolic and cardiovascular challenges of obesity, plus the loss of physical functioning and ability to perform activities of daily living associated with decreases in skeletal muscle mass. As with the previous sub-groups, the objective of effective RT programming for obese and sarcopenic obese older adults is to maximize reductions in FM while maintaining or, if possible, increasing LBM. We present details of the 13 studies included in this sub-group analysis in Table 4.

Eight studies of obese/overweight older adults, [10, 15, 16, 26, 28–31] four studies of older adults with sarcopenic obesity (SO), [17, 27, 32, 33] and one mixed study comparing older adults with and without SO [3] were analyzed. Selectorized resistance exercise machines were the most common RT modality employed, and study durations lasted between 8 weeks [16] and 18 months. [29] None of the RT protocols studied in obese and sarcopenic obese adults attained the gold standard of simultaneous reductions in %BF and increases in LBM. Ten studies reported significant changes in %BF with any significant change in LBM. [3, 15, 16, 26–31, 33] One study reported significant improvements in LBM, with no significant change in %BF. [17] Two studies reported no significant improvement in either parameter. [10, 32] These findings clarify the gaps in current knowledge regarding the optimal application of RT toward the overall goal of improved body composition in obese and sarcopenic obese older adults.

### Pharmaceuticals and Dietary Control

We reviewed eight studies involving RT in older adults and an element of dietary control or pharmaceutical application (Table 5). Two studies [16, 28] investigated differences in body composition between individuals engaged in the Dietary Approaches to Stop Hypertension (DASH) diet and individuals engaged in the DASH diet plus RT. Aguilar and colleagues [18] investigated the ergogenic effects of creatine phosphate supplementation on LBM responses to RT, while Kritchkevsky and Shea [34] evaluated the therapeutic impact of pioglitazone on FM losses when RT combined with a structured RT program. Campbell et al. [10] Brochu et al. [15] and Beavers et al. [29] investigated all the effects of caloric restriction vs. caloric restriction plus RT on measures of body composition. At the same time, Verrieijin and colleagues [31] evaluated the degree to which a high-protein diet can attenuate LBM losses in obese older adults participating in a structured RT program while following a hypocaloric diet.

The finding by Beavers et al. [35] that dietary restriction plus AT led to a significant loss in LBM (− 1.6 + 0.3 kg, − 3.1%) compared to dietary restriction plus RT (− 1.5%, *p* = 0.02) and dietary restriction alone (− 2.0%, *p* = 0.02) underscores what is known about the importance of RT as a mechanism to mediate LBM losses during periods of WL. It demonstrates how all exercise-induced reductions in BM cannot be considered equal in terms of the overall benefit to health. Results of the caloric restriction plus RT studies [10, 15, 29] also highlight the existence of a ceiling-effect concerning the ability of RT-alone to facilitate healthy bodyweight changes. The finding that creatine supplementation led to significantly greater increases in LBM compared to the placebo in older adults after a period of RT [18] has apparent implications for future study in this population, given their susceptibility to developing sarcopenia and, in general, slowing the reductions in muscle mass and strength known to occur with age. Pioglitazone increased visceral fat loss, while the RT element of the study was credited with facilitating the attenuated reductions in skeletal muscle mass. [34] Overall, however, additional research is needed to elucidate more information about sex differences and how its impact on body composition influences functional status. [34]

## Discussion

This scoping review characterized the existing literature on RT and weight loss and body composition changes in healthy older adults, healthy older women, older adults with obesity and sarcopenic obesity and the effect of various dietary and therapeutic approaches. The purpose of this study was to provide a survey of existing literature on the topic, particularly regarding the degree of similarity in methodological approaches used and outcomes reported. Given the limited high-quality evidence we found showing that resistance training (RT) coupled with a healthy diet results in substantial fat loss and lean muscle gain, it may be too early to label our findings as an evidence-based strategy for weight loss through RT in older adults. The main findings and recommendations are summarized below.

### RT Protocols Used for Weight Loss in Older Adults

The main takeaway message from this review is that the true value of RT as it relates to WL is not that it leads to more significant reductions in total BM than other exercise approaches such as AT or circuit resistance training, but rather that it is an adjunct modality leading to the *right kind* of weight loss, namely reductions in FM only. As explained throughout this review, older adults are already facing a decline in their skeletal muscle mass and strength, which is critical for these individuals to maintain as they age. The pursuit of body weight reductions via exercise should undoubtedly be recommended to obese and sarcopenic obese older adults. However, consideration of the form said exercise should take is of critical importance lest individuals inadvertently trade reduction in one age-related chronic disease (obesity) for the accelerated onset of another (sarcopenia). Bodyweight reductions through exercise are undoubtedly an essential objective for obese older adults. Higher exercise intensities and training volumes facilitate more significant improvements in LBM. [8, 27] However, they also tend to require specific exercise machines located in a fitness facility and a level of supervision for many older adults. Lower-intensity modalities such as body weight, resistance bands and hand weights may not facilitate the same level of improvement in muscular size and strength but have the benefit of being inexpensive, portable and easy to use.

### Older Women

Women are at greater risk for age-related reductions in muscle mass and strength than men [21, 22], meaning there is added importance to ensuring exercise modalities that they engage in and are recommended to them take into consideration the need to maintain LBM. Studies reviewed indicate the existence of multiple suitable approaches in this regard [3, 8] and the task of exercise professionals is to translate this knowledge out of academic journals and into the broader population. The impact of RT on positive changes in body composition in older women seems greatest at higher intensities and when selectorized exercise machines are used. Exploring ways to make members of this population group comfortable with the idea of regularly attending an exercise facility for their RT exercise, rather than doing it at home with low-complexity modalities such as body weight and elastic bands, may be worthwhile. In time, recommendations may expand to include using ergogenic aids such as creatine and pioglitazone.

### Role of Diet in Achieving Healthy Body Weight Changes

A consequence of a detailed analysis of RT and the most effective implementations to effect positive body composition changes is that it can be easy to lose sight of the fact that exercise is only one-half of the equation necessary to achieve maximum results in this domain. Multiple studies in this review remind us that regardless of how effective particular RT approaches may be at facilitating healthy body composition changes, there is an upper limit to their effectiveness and that proper dietary habits must also be present to obtain the greatest possible result for changes in body weight. Beavers et al. [35] showed that even an elevated protein intake and regular RT participation cannot wholly attenuate the loss of LBM in the presence of a hypocaloric diet. Explained broadly, the concept of anabolic resistance refers to older adults’ decreased responsiveness of muscle tissue to anabolic stimuli leading to decreased muscle mass and strength and a reduced ability to repair and build new muscle tissue. [42] In addition to regular structured resistance exercise, daily protein intakes of approximately 1.6 g/kg of body weight may be necessary to overcome this additional challenge of aging. [42–44]

### Feasibility and Tolerability of RT as an Exercise Modality to Promote Weight Loss

None of the studies retained for analysis directly commented on the feasibility or tolerability of their protocol. However, several conclusions can be drawn from the studies reporting the highest dropout rates. In the study by Chen et al. [33], only 60 of the 93 participants enrolled at baseline completed the final post-test (36% dropout rate). Aside from a single sentence in which the authors indicate reasons for discontinuation included loss of motivation, family factors, and difficulty in time arrangement, no further discussion was provided, limiting the degree to which valuable lessons from their experience can be carried forward. Reasonable speculation may be that the scheduled four-week interval between completion of the final exercise session and the first post-testing session reduced the ultimate number of participants who completed the study.

Bardstu and colleagues [13] recorded a 44% dropout rate across their 8-month RT in older adults. The authors indicated the rate may have been partially due to the higher median age of their participants (86.0 years) relative to the vast majority of exercise studies in older adults, where > 65 years is used as the standard minimum enrollment age. The authors attributed the high observed dropout rate partly to their participants' advanced age and low health status and suggested that future studies should include strategies aimed at maximizing compliance, such as strengthening older adults' self-efficacy and motivation. The authors also suggested that future research should also evaluate the effect of earlier implementation and the cost-effectiveness of implementing long-term RT in older adults' real-life settings.

An additional key area of concern for the future is how the importance of WL with minimal FFM loss can become more widely known in medicine, public health, and in the general population. We propose four possible ways this may be achieved: (1) Education and awareness: By educating healthcare professionals, public health advocates, and the general population about the dangers of losing muscle mass during weight loss, people can be made aware of the importance of preserving muscle mass while losing weight. This can be done through health seminars, workshops, and public health campaigns; (2) Promoting healthy weight loss strategies: Encouraging the use of healthy weight loss strategies, such as a balanced diet and regular exercise, can help to minimize muscle loss during weight loss. This can be done through partnerships between public health organizations and nutrition and fitness experts; (3) Media coverage: Encouraging media outlets to report on the importance of weight loss with minimal muscle loss can help to raise awareness of this issue. This can include articles in health and fitness magazines and news segments on television and radio; (4) Government involvement: Governments can promote weight loss with minimal muscle loss by funding research and public health initiatives focused on this issue. They can also encourage health insurance companies to cover treatments and interventions that prioritize the preservation of muscle mass during weight loss.

### Recommendations and Future Directions

This review highlights the need for more comprehensive research on the combined effects of resistance training (RT) and a healthy diet for significant fat loss and lean body mass gain in older adults. Ideal future studies should incorporate systematic integration of RT and dietary interventions and include rigorous monitoring of participants' dietary adherence and exercise regimens. It is essential to emphasize a well-rounded evaluation of body composition changes, extending focus beyond weight loss to include preservation and enhancement of lean body mass. Addressing the high attrition rates observed in some studies, the development of feasible and tolerable RT protocols for older adults is crucial. Potential strategies could involve tailored exercise programs, motivational support, and addressing logistical issues that could hinder adherence. Future ideal studies also should focus on reducing heterogeneity in RT methods and body composition evaluation measures, though we acknowledge the challenging practicalities of standardizing practices across this research field.

Two promising areas for future research, illuminated by this review, include the development of RT protocols that effectively increase lean body mass while reducing fat mass in obese and sarcopenic obese older adults. Additionally, there is potential merit in exploring the use of supplements, such as creatine monohydrate, as an ergogenic aid for older adults participating in RT. This is based on sound theoretical rationale and a significant body of preliminary research, providing a robust foundation for future exploration.

## Conclusion

The purpose of this scoping review was to identify and characterize existing research on the use of RT exercise interventions as a modality to promote weight loss in older adults with the end goal of knowledge translation and recommendation to further areas of study. While it is clear that changes in body composition are a crucial outcome measure for this population, the current literature provides limited evidence demonstrating that RT, alongside a healthy diet, results in significant fat loss and lean body mass gain. Furthermore, high attrition rates in some of the analyzed studies highlight the need for well-structured and feasible protocols. Thus, the endorsement of a specific 'evidence-based approach' is premature at this stage. Our findings underline the need for more rigorous, adequately powered studies to explore these relationships further. We aimed to describe populations that have been studied, how RT has been applied, what can be deduced about the feasibility and tolerability of RT used in said manner, what primary outcomes have been addressed and what gaps in the current knowledge have become evident. The two most important takeaway messages we hope to convey from this review are an understanding of why WL changes alone are an incomplete and problematic outcome measure to use with an older population and why changes in body composition (ratio of fat mass to lean body mass) are the appropriate measure to consider for these individuals.

## Data Availability

The datasets used and/or analyzed during the current study are available from the corresponding author on reasonable request.

## Abbreviations

1RM: One-repetition maximum
ADP: Air-displacement plethysmography
AT: Aerobic training
BF: Body fat
BIA: Bioelectrical impedance analysis
BM: Body mass
BMI: Body mass index
CH: Care home
CINAHL: Cumulative Index to Nursing and Allied Health Literature
CR: Caloric restriction
CRP: Creatine phosphate supplementation
CT: Combined training
D: Drug trial
DASH: Dietary approaches to stop hypertension
DEXA: Dual X-ray absorptiometry
ES: Effect size
FFM: Fat-free mass
FM: Fat mass
H: Healthy
HIT: High intensity interval training
HRT: Heavy resistance training
LBM: Lean body mass
LMM: Lean muscle mass
MRT: Moderate intensity resistance training
NPRT: Non-periodized resistance training
NR: Not reported
NPRT: Non-periodized resistance training
O: Overweight or obese
P: Postmenopausal
PRT: Periodized resistance training
QE: Quasi-experimental
RCT: Randomized-controlled trial
RT: Resistance training
SD: Standard deviation
SO: Sarcopenic obesity
W: Women only
WL: Weight loss
WPS: Wide pyramid system
